# Physicochemical, Mechanical, and Histological Assessment of Explanted Polypropylene Meshes from Recurrent Abdominal Wall Hernia Repairs

**DOI:** 10.3390/ma19143048

**Published:** 2026-07-15

**Authors:** Olga A. Legonkova, Tatyana I. Vinokurova, Viktoria V. Stafford, Dmitriy I. Kopitsyn, Badri S. Gogia, Galina A. Davydova, Egor V. Kasatkin, Mariya I. Styazhkina, Rifat R. Alyautdinov, Vladimir A. Vinokurov, Victoria Yu. Grigorieva, Veronica Manescu (Paltanea), Iulian Vasile Antoniac, Julietta V. Rau

**Affiliations:** 1 A.V. Vishnevsky National Medical Research Center of Surgery, Ministry of Health of the Russian Federation, Bolshaya Serpukhovskaya St. 27, Moscow 117997, Russia; legonkova@ixv.ru (O.A.L.); vinokurova@ixv.ru (T.I.V.); v.v.stafford.vity@mail.ru (V.V.S.); gogia@ixv.ru (B.S.G.); egor.kasatkin.2020@mail.ru (E.V.K.); mari-stna@mail.ru (M.I.S.); alyautdinov@ixv.ru (R.R.A.); 2 All-Russian Research Institute of Experimental Veterinary Medicine Named After K.I. Scriabin and Y.R. Kovalenko (Federal Scientific Center), Russian Academy of Sciences, Ryazansky Prospekt 24/1, Moscow 109428, Russia; 3 Department of Chemical Technology and Ecology, Gubkin Russian State University of Oil and Gas (National Research University), Leninsky Prospekt 65, Moscow 119991, Russia; kopicin.d@inbox.ru (D.I.K.); vladimir@vinokurov.me (V.A.V.); 4 Institute of Theoretical and Experimental Biophysics, Russian Academy of Sciences, Institutskaya St. 3, Pushchino, Serpukhov District, Pushchino 142290, Russia; davidova_g@mail.ru; 5 Institute of Pharmacy, Department of Analytical, Physical and Colloid Chemistry, Sechenov First Moscow State Medical University, Trubetskaya St. 8, Build. 2, Moscow 119048, Russia; grigoreva_v_yu@staff.sechenov.ru; 6 Faculty of Material Science and Engineering, National University of Science and Technology Politehnica Bucharest, 313 Splaiul Independentei, District 6, RO-060042 Bucharest, Romania; veronica.paltanea@upb.ro; 7 Faculty of Electrical Engineering, National University of Science and Technology Politehnica Bucharest, 313 Splaiul Independentei, District 6, RO-060042 Bucharest, Romania; 8 Academy of Romanian Scientists, 54 Splaiul Independentei, RO-050094 Bucharest, Romania; 9 Istituto di Struttura della Materia, Consiglio Nazionale delle Ricerche (ISM-CNR), Via del Fosso del Cavaliere 100, 00133 Rome, Italy; giulietta.rau@ism.cnr.it

**Keywords:** explanted endoprostheses, hernia recurrence, mesh endoprostheses, polypropylene, deformation-strength properties, differential scanning calorimetry, IR spectroscopy, histology

## Abstract

The purpose of this study is to investigate the physical, chemical, and structural characteristics of 13 polypropylene mesh endoprostheses explanted due to hernia recurrences across an in vivo timeline ranging from 1 to 14 years, as well as the morphology of the surrounding tissues, to assess the possible influence of polymer degradation on explantation timing. Physical and mechanical testing, gas chromatography–mass spectrometry, differential scanning calorimetry, infrared spectroscopy, and histological assessment of tissue response to the implanted endoprostheses were employed. The tensile strength and elongation measurements of the explanted meshes remained within functionally acceptable clinical ranges, showing no statistically significant decline compared to baseline parameters. Polypropylene monofilaments of mesh endoprostheses were subjected to surface changes in the form of transverse cracks during exposure to patient tissues; however, the depth of these cracks was self-limiting (confined to 3–7% of the filament diameter) and did not affect the bulk strength properties of the endoprostheses. Histological examination demonstrated a chronic foreign body response that transitioned from early focal necrosis to a stable tissue capsule. While these data suggest that superficial micro-cracking did not markedly compromise bulk mechanical performance over the 14-year span, localized physical or biological tissue–implant interactions could still contribute to structural instability, meaning polymer degradation cannot be definitively ruled out as a contributing factor to recurrence in all clinical cases.

## 1. Introduction

With the development of plastic surgery, the problem of eliminating extensive defects of the anterior abdominal wall, followed with hernia repairs, tumor resections and the formation of cutaneous muscular abdominal flaps, has become particularly relevant. In recent years, the use of endoprostheses has become widespread both internationally and in Russia, providing more reliable reinforcement of the abdominal wall and reducing the incidence of hernia recurrences.

While early mesh endoprostheses were manufactured from materials such as polyethylene terephthalate (PET) [1,2,3,4], polypropylene (PP) monofilament meshes (Marlex Mesh, Prolene Mesh, Premilene Mesh, SurgiMesh, Esfil, etc.) have served as the clinical “gold standard” for over 60 years [5,6,7], supplemented by newer alternatives including polyvinylidene fluoride (PVDF) (PVDF Mesh, Uniflex) [8], polytetrafluoroethylene (PTFE) [9], and composite materials [10,11,12].

Despite progress in the surgical treatment of hernias, postoperative complications in the form of recurrences occur both in laparoscopic procedures, where complication frequency reaches 20%, and up to 35% in high-risk groups [13], and in conventional techniques, when recurrence rates may reach 48% [14,15]. For instance, the rate of recurrence after inguinal hernia repair with the use of mesh endoprosthesis reaches up to 13% for all hernioplasties worldwide.

To systematically address these clinical outcomes, it is critical to clearly distinguish between the diverse, multifactorial etiologies driving hernia recurrence, which can be categorized into distinct pathways: (i) material degradation of the polymer matrix; (ii) surgical technique and fixation failures, such as technical errors in implant anchoring or incorrect tensioning [16,17,18,19,20,21,22,23,24,25]; (iii) mechanical implant performance, which includes mesh shrinkage and structural displacement over time [17,26]; (iv) biological complications, such as a high vulnerability to localized infection [2,3,4]; and (v) patient-related risk factors, including obesity, diabetes mellitus, smoking, and native connective tissue dysplasia [14,15,18,19,20,21,22,23,24,25]. Superimposed upon these clinical variables is the unresolved core scientific question: whether long-term in vivo exposure causes clinically meaningful degradation of the polypropylene polymer matrix itself, thereby progressively compromising its structural safety, or whether the polymer remains functionally stable while other surgical and patient-specific factors dictate long-term success.

The literature presents diametrically opposite opinions regarding the causes of in vivo surface changes in PP meshes that can be easily divided into two main hypotheses as follows. The first one is related to surface degradation generated by the bio-oxidative processes, which occur due to host foreign body reactions [27,28,29,30,31,32,33]. The scientists who support this theory argue that reactive oxygen species (ROS) and hydrolase enzymes secreted by the giant foreign body cells cleave the amorphous polymer chains, leading to significant surface erosion, a possible mechanical failure, and the embrittlement of the implant [33,34,35]. On the other hand, the second hypothesis is based on the presumption that the PP remains chemically inert in in vivo conditions, so the visible surface cracks are physical artifacts of the well-known environmental stress cracking or are due to the internal manufacturing stresses induced by some implant preparation methods [36,37] rather than the classical chemical degradation. By analyzing the literature, one can immediately notice that these divergences are confounded by the differences in sample sources and restricted implantation spans. For example, some studies investigating important, progressive degradation use animal models over weeks to months [35], whereas others are limited to human cohorts with a maximum time span of 5 years [36]. Conversely, investigations claiming complete polymer stability lack multi-scale analytical validation, bulk thermal analyses, or cross-sectional microstructural mapping [28,31]. However, an important research gap in the long-term evolution of PP meshes over extended clinical time periods has been noted. Also, it is unclear whether surface microstructural changes progressively influence the bulk mechanical properties or remain safely within safe limits over time.

**Figure 1:**
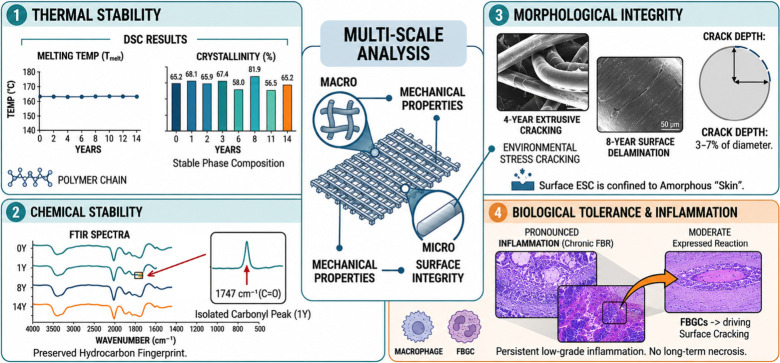
Schematic representation of the study.

Based on the literature data, it can be concluded that there are some uncertainties regarding the mechanism of polypropylene endoprosthesis degradation in vivo. That is why the objective of this study is to summarize our own data on the behavior of PP mesh endoprostheses explanted after different terms of use. The purpose of this study is to investigate the physical, chemical, and structural characteristics of polypropylene mesh endoprostheses explanted due to hernia recurrences across a long timeline spanning up to 14 years, as well as the morphology of tissues surrounding the stent to assess the possible influence of destructive changes in polypropylene on the timing of explantation (Figure 1).

## 2. Materials and Methods

### 2.1. Materials

The explanted endoprostheses consisted of PP mesh endoprostheses (hereinafter referred to as PP endoprostheses) from various manufacturers, differing in structure and duration of residence in patient tissues (from 1 year to 14 years) following surgical interventions for recurrent abdominal wall hernias. Explanted endoprostheses were treated to remove connective tissue using 10% potassium hydroxide (KOH) solution (Merck KGaA (Sigma-Aldrich), Darmstadt, Germany) at 40–50 °C until achieving complete removal of biological material. On average, this process took from 30 min to 1 h, depending on the amount of biological tissue present on the explanted PP endoprosthesis. This specific alkaline cleaning protocol was selected because polypropylene is an aliphatic hydrocarbon polymer characterized by extreme chemical resistance to strong bases, meaning exposure to 10% KOH at 40–50 °C does not induce structural degradation, swell the polymer matrix, or alter the intrinsic SEM-visible surface topography. Furthermore, while this treatment effectively hydrolyzes and removes adherent biological soft tissues to expose the underlying mesh structure, it does not strip away or modify chemically bound, native in vivo oxidation products (such as carbonyl groups incorporated into the polymer backbone).

All the collected samples exhibit a warp-knitted monofilament weave structure. The explanted samples were obtained during revision surgeries at multiple clinical centers over a lengthy period, making it impossible to verify the original product types and specific manufacturers from the patients’ medical records. To address this variability and facilitate systematic analysis, the meshes were categorized into standardized clinical weight grades based on their measured surface density (*M_s_*). The classification is as follows: ultra-lightweight (*M_s_* < 35 g/m^2^), lightweight (35 ≤ *M_s_* < 50 g/m^2^), medium-weight (50 ≤ *M_s_* < 80 g/m^2^), and heavyweight (*M_s_* ≥ 80 g/m^2^) (Table 1). The inability to identify the specific commercial manufacturers and proprietary processing histories represents an inherent limitation of this retrospective explant cohort. The investigated sample cohort contained 12 meshes harvested from 12 individual patients (excluding the first sample—unimplanted baseline mesh). These patients underwent an open or laparoscopic surgical procedure for midline ventral or incisional recurrent abdominal wall hernias. As a direct consequence of the retrospective, multi-center nature of our long-term tracking timeline of 14 years post-implantation, detailed patient-specific recording related to surgical fixation methods, sub-classification of the anatomical mesh positioning (onlay, pre-peritoneal, and retro-muscular), and additional patient comorbidities could not be found across the archival hospital repositories. To control for potential clinical cofounding variables despite data gaps, we applied the following strict inclusion criteria at the time of retrieval: (i) all explants were exclusively taken from recurrent incisional or ventral hernias of the anterior abdominal wall; (ii) mechanical hernia recurrence served as the primary and uniform clinical indication for explantation; and (iii) cases presenting with infected surgical sites or a history of chronic mesh-related infections were systematically excluded from this study. This approach ensured that the material alterations observed represent a clean, host-mediated bio-stability profile over a long-term in vivo period.

Despite the inherent variations in baseline weight grades, structural densities, and unknown manufacturing origins, all samples displayed the same self-limiting degradation pattern. Notably, micro-cracking did not extend beyond the outer 3% to 7% amorphous “skin” of the monofilaments. This observation confirms that long-term bulk stability is an intrinsic physical characteristic of medical-grade drawn polypropylene monofilaments in vivo, regardless of the specific commercial supplier.

### 2.2. Methods

Determination of deformation-strength characteristics of PP meshes was performed with the help of the EZ-LX-0.5 universal testing machine (SHIMADZU, Kyoto, Japan). Considering that explanted endoprosthesis samples varied in shape and size, tests were conducted on specimens 15 mm wide with a grip length of 20 mm. In the case of each distinct explanted mesh, a total of five independent replicates were harvested and tested (*n* = 5) to generate statistically reproducible mean values. All mechanical measurements were conducted under standard laboratory conditions, specifically at 23 °C and 50% relative humidity. The mesh strips were tested in a dry state immediately after the chemical tissue extraction protocol. To consider the anisotropic mechanical behavior of warp-knitted architectures, the specimen strips were systematically cut and oriented parallel to the longitudinal warp direction of the knit. Strips were selected exclusively from clear, non-deformed macro-zones of the recovered mesh sheet, avoiding peripheral edges that had sustained direct damage from the scalpel or forceps during the surgical revision procedure. Uniaxial tensile loading was applied continuously at a constant crosshead speed of 50 mm/min until catastrophic fracture of the specimen occurred. To compare strength characteristics among endoprostheses, the breaking load per 1 cm of specimen width (*p*, N/cm) was calculated.

Measurement of thermophysical properties of mesh endoprostheses was evaluated with the help of differential scanning calorimetry (DSC). DSC thermograms were obtained via the DSC 214 Polyma instrument (NETZSCH, Selb, Germany) under a nitrogen atmosphere. A 5–6 mg sample was placed into an aluminum crucible, then two scans in dynamic mode at a heating rate of (ν ± 10) K/min over a temperature range of −20 to +200 °C were done.

The degree of crystallinity (*DC*) (%) was calculated using Equation (1):

*DC* = (Δ*H*/Δ*H*_100%—cryst_)∙100%, (1)

where Δ*H* is the enthalpy of sample melt (the 2nd scan) and Δ*H*_100%—cryst_ is the melt enthalpy of the 100% crystalline sample, equal to 138.0 J/g [36].

Investigation of surface changes was performed via attenuated total reflectance (ATR) spectroscopy in the wavenumber range of 7800–350 cm^−1^ on an IR Spirit FTIR spectrometer (Shimadzu, Kyoto, Japan). The IR spectrum of pure PP from the instrument library was used as a reference. The degree of crystallinity (*DC_IR_*) was calculated using Equation (2) [37]:

(2) $${\mathrm{DC}}_{\mathrm{IR}}\;=\;109\cdot \frac{D_{998}-D_{908}}{D_{972}-D_{908}}$$

where *D_xxx_* represents optical density at wavelengths 998, 972, and 908 cm^−1^, respectively.

To determine the possible mechanism of biodegradation of PP implants, a Hewlett Packard HP-6890 gas chromatograph (Hewlett Packard, Wilmington, DE, USA) with a mass-selective detector was used. A total of 1.0 mL of acetonitrile (Merck KGaA (Sigma-Aldrich), Darmstadt, Germany) was added to 200 mg of the sample, and the mixture was treated for 30 min in an ultrasonic bath. A total of 0.2 mL was taken from the resulting extract and evaporated to dryness under nitrogen stream, and 0.05 mL of BSTFA (*N*,*O*-bis-trimethylsilyltrifluoroacetamide) solution (Merck KGaA (Sigma-Aldrich), Darmstadt, Germany) in acetonitrile (volume ratio 1:4) was added. The sample was heated in a thermostat for 20 min at 80 °C. Both non-polar and polar extractants (hexane, acetonitrile) were used in the study. It was established that the most informative results were obtained when derivatizing acetonitrile extracts via silylation with BSTFA.

Investigation of the explanted mesh samples’ surfaces was conducted via Jeol JCM-6000 Plus scanning electron microscope (JEOL Ltd., Akishima, Japan).

Surface density (*M_s_*, g/m^2^) of endoprostheses was determined gravimetrically according to GOST 8845-87 [38]. Thickness and diameter of explanted endoprostheses were measured according to GOST 9696-82 [39].

Histological preparations were prepared via paraffin filling of the tissue samples fixed in 10% formalin solution (BioVitrum, Saint Petersburg, Russia). The following laboratory equipment were used: an STP 120 carousel-type processing unit (Thermo Scientific, Dreieich, Germany), EC 350-1 pouring apparatus with EC 350-2 cryo-console (Thermo Scientific, Dreieich, Germany), Microm HM325 rotary microtome (Thermo Scientific, Germany), and heating stage (Medax, Neumünster, Germany). Paraffin blocks with 5 µm thickness were cut, colored with hematoxylin and eosin, and covered with coverslips using SubX medium (Leica, Wetzlar, Germany).

Statistical analysis of the results was performed using STATISTICA for Windows software, version 10.0 (StatSoft Inc., Tulsa, OK, USA). Descriptive statistics were calculated for all measurable experimental parameters, with data presented as mean values plus or minus standard deviation. This retrospective study evaluates a specialized cohort of long-term human explants collected over a span of 14 years from multiple clinical centers, with unknown manufacturing origins. Therefore, the data were analyzed primarily using descriptive statistics and comparative trend mapping. No formal inferential statistical tests, such as ANOVA, Student’s *t*-test, or post hoc comparisons, were conducted between specific time intervals due to unequal sample sizes among weight grades and the lack of a standardized, homogeneous distribution across commercial suppliers within the time categories. This descriptive approach ensures that the physical properties are interpreted within their specific clinical context without introducing misleading statistical significance.

## 3. Results and Discussion

### 3.1. DSC Results

To evaluate the stability of PP endoprostheses over time under the influence of the biological environment, their thermophysical properties were investigated. These data at different explantation time intervals are presented in Table 2.

Typically, during polymer degradation, thermophysical parameter values change, allowing for indirect assessment of the extent of various alterations. For instance, the degree of crystallinity determines the strength, rigidity, temperature resistance, and chemical stability of polypropylene. For an endoprosthesis, it is critical to preserve its shape and avoid degradation within the body. In our case, the thermograms exhibited identical characteristics, each containing a single melting peak with values for all PP samples, regardless of implantation duration or manufacturer, falling within the range of 162–166 °C; the degree of crystallinity across the entire cohort ranged from 56.5% to 81.9% (Table 2). Due to the inherent heterogeneity of these clinical samples, which are not matched by original manufacturer or specific initial mesh macro-structure, these thermal metrics must be interpreted with caution. By comparing explanted PP samples, an important resilience in thermal properties, despite differences in structural design and provenance from various manufacturers, was observed over the in vivo timeline of up to 14 years. Also, the DSC data showed that the crystallization and melting temperatures fluctuated minimally within the ranges of 104 °C to 107 °C and 162 °C to 166 °C, respectively. The explanation is that the PP filaments do not undergo significant thermodynamic rearrangement or intense bulk thermal degradation over the investigated clinical times. However, for sample 13 (14-year mark), the melting point (164 °C) and the degree of crystallinity (62.5%) are almost identical to the baseline sample (sample 1), and it could be concluded that the preservation of thermal PP properties underscores the long-term inert behavior of the polymer within in vivo conditions.

In addition, the non-linear fluctuations in the enthalpy (113 J/g) and degree of crystallinity (81.9%) values, which characterize sample 10 (8-year mark) in Table 2, should be closely investigated. It is well known that, in the case of polymeric materials, chemi-crystallization is the main mechanism that determines an increase in crystallinity degree. This process is present when oxidative stress from the patient’s foreign body response selectively breaks down some of the amorphous chains. This phenomenon allows the liberated segments to be realigned into a crystalline lattice structure. Further, since different manufacturers produced these explanted PP samples, this anomalous peak can be verified by considering the variable spinning residual stress introduced during the industrial extrusion and high-ratio drawing processes of different production lines. High residual orientation and spinning stresses can significantly accelerate local post-crystallization phase transitions or yield a higher baseline crystallinity state independently of the implantation duration.

Similarly, the isolation of a carbonyl group (-C=O at 1747 cm^−1^) exclusively within the 1-year sample (sample 2) correlates tightly with the patient’s localized acute-on-chronic inflammation status rather than bulk chemical decay. As demonstrated in our histological analysis, early-stage explants exhibit localized tissue necrosis, marked hemorrhagic zones, and intensive foreign body giant cell grouping. This aggressive local inflammatory microenvironment releases a concentrated amount of ROS capable of inducing transient surface oxidation. Once this outer, superficially oxidized layer undergoes micro-cracking and subsequent mechanical delamination due to abdominal wall motion, the pristine, hydrophobic bulk polymer core is re-exposed. Supplementary to this, the value of crystallinity (computed based on Equation (1)) being almost equal to that of the baseline level (56.5 at 11 years (sample 11) and 65.2 at 14 years (sample 13)) supports the conclusion that while surface oxidation and remodeling occur, the structural integrity of the PP endoprostheses is stable over about 10 years post-implantation.

### 3.2. IR Spectroscopy Results

**Figure 2:**
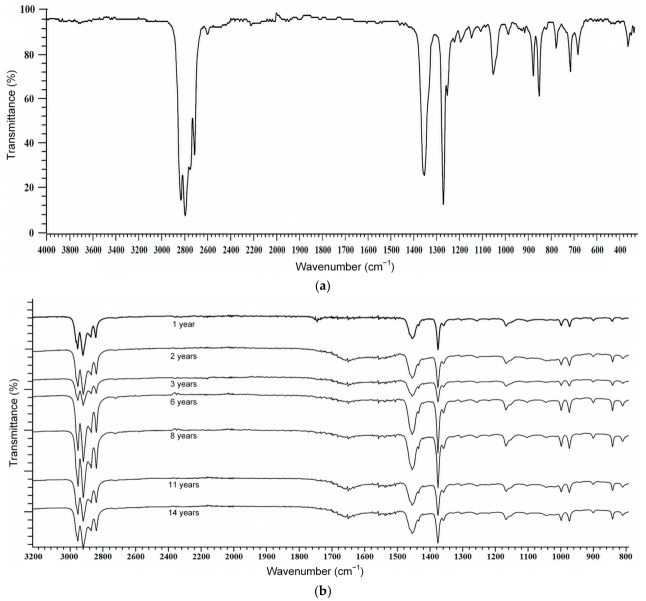
IR spectra of PP meshes. (**a**) Reference IR spectrum of pure PP (from Shimadzu instrument database); (**b**) IR spectra of meshes depending on implantation duration (1, 2, 3, 6, 8, 11, and 14 years from upper to lower curves).

IR spectra of the surfaces of the original PP and explanted samples are shown in Figure 2.

The IR spectra obtained for the explanted mesh samples once again demonstrated high chemical stability, preserving the classic spectroscopic features of the PP pristine baseline. The main absorption bands specific to aliphatic hydrocarbons will be detailed. The intense doublet/triplet cluster between 2800 cm^−1^ and 3000 cm^−1^ corresponds to asymmetric and symmetric C-H stretching vibrations of methyl (-CH_3_) and methylene (-CH_2_-) groups, while the important bending vibrations at approximately 1455 cm^−1^ (-CH_2_-Fbending) and 1376 cm^−1^ (-CH_3_ symmetric mode) remain unaltered across the samples. IR analysis could clearly identify the chemical groups of subsurface and surface filaments. The absence of significant peak shifts or baseline distortions in the sample spectra demonstrated that hydrophobic hydrocarbon chains are resistant to macromolecular reorganization or chemical attack, as observed during implantation for more than 10 years.

It is worth mentioning that the presence of the carbonyl (-C=O) stretching band in the 1700 cm^−1^ and 1750 cm^−1^ interval highlights the fact that the reactive oxidative species initiate oxidative degradation under macrophage cell mechanisms. In our investigation, we have identified a single carbonyl peak at about 1747 cm^−1^ in sample 2 (1-year-old mark), which is absent in all other long-term implantation samples. It could be foreseen that polymer oxidation is neither linearly progressive nor inevitable under in vivo conditions. It is assumed that the localized oxidation process was strongly influenced by the patient’s condition, such as a localized acute inflammatory zone, or by variations in the producer’s stabilization procedure. However, the PP meshes explanted after 11 years and 14 years do not exhibit this detectable carbonyl group. While this indicates that no clear evidence of progressive, long-term oxidation was detected under the analytical conditions used, it should be noted that the absence of a distinct peak does not conclusively prove complete chemical safety. The attenuated total reflection IR samples have specific surface interaction zones and may lose highly localized or scattered micro-pockets of polymer oxidation. Furthermore, as a limitation of explant processing, the preceding 10% KOH treatment could potentially dissolve or remove loosely bound, low-molecular-weight oxidized species from the superficial cracks. To check this evolution more quantitatively, a relative carbonyl index (*CI*) was computed as the ratio of the intensity of the carbonyl band (1747 cm^−1^) to the reference aliphatic C-H bending band (1455 cm^−1^).

(3) $$CI=\frac{I_{1747}}{I_{1455}}.$$

In the case of the 1-year sample (sample 2), the calculated *CI* value was minimal, at 0.041. In contrast, for all other long-term explants, the index value essentially decreased to zero. This quantitative measure reinforces the conclusion that the progression of the surface-bound oxidation layer is significantly limited over longer implantation periods, rather than penetrating deeper into the material’s core. On the other hand, the IR method is used to evaluate the morphological phases of the polymer, such as crystalline or amorphous phases. In our case, the computed IR crystallinity values (*DC_IR_*), ranging from 62% to 73% for all samples (computed based on Equation (2)), are fully consistent with the crystallinity values obtained from DSC analyses (56.5% to 81.9%). It can be immediately concluded that a disordered amorphous layer on the surface does not form in the long-term explants, indicating that no surface-specific degradation layer is formed. Overall, the DSC and IR testing methods confirm that the PP meshes maintained phase integrity throughout the extended clinical time period. It should be noted that the comparability of crystallinity values obtained by different methods suggests that, firstly, either method may be used to calculate this parameter, and secondly, no critical changes occur on the surface of PP endoprostheses over a 14-year period.

### 3.3. SEM Results

**Figure 3:**
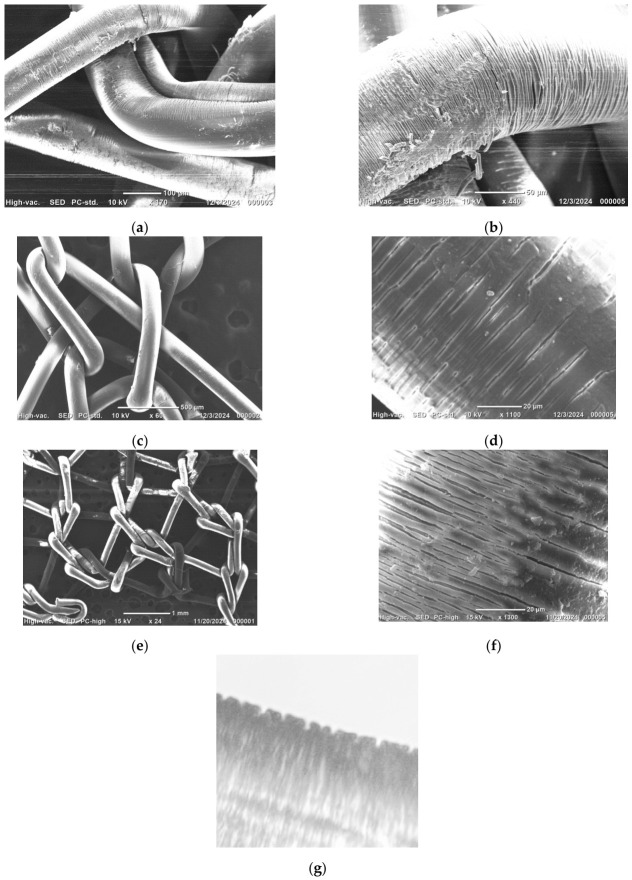
(**a**,**b**) show endoprosthesis after 4 years in vivo (sample No. 7), with magnification of ×170 and ×440, respectively; (**c**,**d**) show endoprosthesis after 8 years in vivo (sample No. 10), with magnification of ×60 and ×1100, respectively. SEM micrographs of PP monofilaments in endoprostheses. (**e**,**f**) show polypropylene mesh component of composite endoprosthesis after 14 years in vivo (sample No. 13), with magnification of ×60 and ×1300, respectively; (**g**) shows polypropylene monofilament after 11 years in vivo (sample No. 11).

Scanning electron microscopy (SEM) investigations of PP endoprosthesis surfaces revealed the presence of cracks on the surface of PP monofilaments (Figure 3). Alongside these changes, unaltered areas of the mesh were also observed.

The SEM investigation shows spatial evidence of the so-called physical remodeling that is present only at the living tissue–prosthesis boundary. As it could be easily noticed in the case of explants (4-year mark, Figure 3a,b, to 14-year mark, Figure 3e,f), the PP monofilament’s surface exhibits a morphological modification characterized by the existence of micro-cracks and superficial delamination. This observed degradation pattern represents a highly plausible proposed mechanism—namely, environmental stress cracking—which is likely driven by the synergy between continuous mechanical stress arising from the physiological mechanics of the abdominal wall and chemical attack. This proposed pathway suggests that the human body’s foreign response activates immune system cells to adhere to the PP mesh and secrete ROS, as previously explained, as well as hydrolytic enzymes. This localized factor is hypothesized to act on disordered tie-chains placed at the fiber surface. As these polymeric chains cleave, manufacturing residual stresses are released, potentially forming regular arrays of transverse and longitudinal micro-cracks.

From the high-magnification images (Figure 3d,f,g), it is obvious that the structural degradation is present only in the outermost shell of the fiber. From the cross-sectional view (Figure 3g) obtained for the 11-year-mark sample, the depth of micro-cracks penetrates only to 3% to 7% of the total monofilament diameter. To verify the methodology across the cohort, we conducted a quantitative cross-sectional micro-measurement using specialized digital imaging software (ImageJ 1.54r, NIH, Bethesda, MD, USA). We randomly sampled and evaluated a total of 50 independent micro-cracks distributed across 15 different monofilament segments, using both cross-sectional and high-magnification views. This systematic measurement confirmed that the boundary restriction of 3% to 7% consistently applies to all analyzed long-term explants. The explanation is that the superficial boundary results from a highly oriented, anisotropic crystalline structure formed during the standard manufacturing process. This hydrophobic, densely packed structure could be considered a barrier against inward diffusion of water, cell-free radicals, and oxygen. The SEM images are in accordance with the DSC analysis because clarifying that the cracking is limited to the outer 3–7% of the shell explains why the melting temperature and overall crystallinity degree are stable for long-term implantation samples. In addition, the surface-confined degradation clarifies the IR results. Since the superficial degraded thin layer readily delaminates or sheds small fragments over time, carbonyl groups do not linearly accumulate on the mesh surface. Instead, they are continuously cleared by the surrounding tissue microenvironment, leaving behind a relatively clean, unoxidized PP core beneath—explaining well why the -C=O peak at 1747 cm^−1^ was only an isolated observation for sample 2 and not a progressive trend. In conclusion, the microscopic investigations of PP mesh endoprostheses explanted after periods of up to 14 years demonstrate that during residence in patients’ biological tissues, the surface of PP monofilaments undergoes alteration, irrespective of its structure or manufacturer. Surface cracking of PP monofilaments appears as early as 1 year in vivo and is observed at all subsequent intervals but only affects the superficial layers of the monofilaments.

### 3.4. Physico-Mechanical Tests Results

Physical and mechanical properties of explanted endoprostheses are presented in Table 3. The required strength of an endoprosthesis is typically determined by intra-abdominal pressure and defect diameter. According to authors [40], the abdominal fascia withstands forces of 60–80 N/cm in the horizontal direction and 20–30 N/cm in the vertical direction. Assuming the abdominal cavity represents a thin hollow sphere, its rupture force is estimated at 4–16 N/cm. Authors of the study [41] demonstrated that, based on a maximum intra-abdominal pressure of 20 kPa, the physiological strength norm of the abdominal wall corresponds to 16 N per 1 cm of specimen width (16 N/cm). These data have guided hernia surgeons worldwide in selecting endoprostheses for abdominal wall defect repair until 2004, when study [42] proposed a somewhat different calculation method and an alternative strength norm for the abdominal wall: 32 N/cm. Nevertheless, up to now the abdominal wall strength norm of 16 N/cm remains the reference standard for evaluating endoprosthesis strength [43,44].

The differences observed in the breaking loads between the loop column (*P_loop_*) and the loop row (*P_row_*) underline the anisotropic character of the PP meshes. It can be observed that, across all samples, the loop column shows higher tensile strength (Table 3). This structural anisotropy is important when the proper alignment of interlocking monofilament loops generates preferential load-bearing pathways. Surgeons make use of these directional properties during hernioplasty by aligning the stronger, stiffer loop columns parallel to the main physiological stress axes of the abdominal wall, which is typically in the transverse direction. Importantly, although these meshes come from various manufacturers and exhibit significant differences in baseline density, both directions consistently maintain strengths well above the physiological intra-abdominal threshold of 16 N/cm to 32 N/cm in the vast majority of cases. A notable exception is found in sample 8 (5-year mark), where the row-direction strength (*P_row_*) slightly falls below the lower clinical reference standard, measuring 13.6 ± 1.36 N/cm. This decrease in structural integrity highlights that, while the overall mechanical strength remains high across the samples, there is significant variability both between samples and in different directions. The collected explants vary in several aspects, including manufacturer origin, base knitting architecture, density, and filament diameters. Therefore, these mechanical fluctuations cannot be solely attributed to progressive chemical aging in vivo. Instead, they represent a combination of initial structural property variance and complex, patient-specific physical responses. This indicates that structural anisotropy, initial design parameters, and individualized tissue integration, rather than systemic material degradation, primarily influences the directional performance of the mesh in vivo.

The breaking strength is predominantly situated above the clinical threshold across the evaluated periods, though notable directional and sample-to-sample fluctuations occur, alongside an important fluctuation in the relative elongation at break being evidenced. For example, the mesh flexibility is characterized by sharp contractions for sample 4 (2 year-mark) (ε*_loop_* = 52%), followed by a compliant character for sample 13 (14 year-mark) (ε*_loop_* = 123% and ε*_row_* = 146%). In a clinical context, the fluctuations observed are directly linked to the host tissue encapsulation of the implant. During the intermediate years following implantation, the dense infiltration of stiff, collagenous scar tissue into the mesh pores limits the geometric mobility of the knit loops, which artificially reduces elongation. Over a decade in vivo, as the surrounding peri-implant capsule undergoes long-term structural organization, thinning, and functional integration with the healthy native fibro-porous tissue, the mesh partially regains its ability to move freely. This results in a more adaptable mechanical profile that helps prevent long-term patient discomfort or “stiff abdomen” syndrome.

Results of differential scanning calorimetry and IR spectroscopy revealed no substantial differences; therefore, it is logical that physico-mechanical characteristics did not undergo significant changes. Also, a good crystalline core and progressive reinforcement by the patient’s natural tissue allow the mesh to maintain an excellent mechanical safety margin for the majority of long-term samples, though individual architectural configurations may exhibit isolated shifts near clinical thresholds.

### 3.5. Histological Analysis

**Figure 4:**
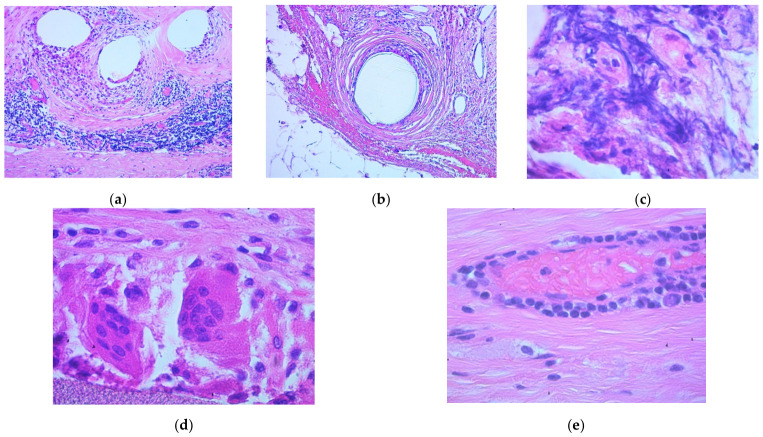
Histological examination. (**a**) is pronounced inflammatory reaction perifocally from PP mesh fragments (sample No. 10, 8 years in vivo), (**b**) is moderately expressed inflammatory reaction (sample No. 13, 14 years in vivo), (**c**) represents area of soft tissue necrosis (sample No. 2, 1 year in vivo), (**d**) is formation of multinucleated foreign body giant cells through perivascular lymphoid cellular reaction. Hematoxylin and eosin staining. Magnification for (**a**,**b**) is 100 and for (**c**,**e**) is 630.

Histological examination of tissues surrounding mesh endoprostheses are summarized for the entire series of samples studied, as no significant differences are observed, and are presented in Figure 4.

The histological pattern is similar regardless of the samples differing in PP endoprosthesis manufacturers and implantation durations. Noteworthy common features of histoarchitectonics include: (1) the foreign body (PP mesh) appears as transparent inclusions with distinct edges, surrounded by a well-organized capsule composed of dense connective tissue fibers and lymphocytes with macrophages; (2) presence of edema in the abdominal wall; (3) variable severity of hemorrhagic areas; inflammatory reactions manifested as lymphocytic infiltration in surrounding tissues; formation of multinucleated foreign body giant cells, which are known to release reactive oxygen species that attack the polymer’s amorphous regions; and deformation of adipocytes at sites of contact between the PP mesh and the patient’s abdominal wall. Additionally, focal soft tissue necrosis may be observed in early explants (e.g., at 1 year), reflecting initial post-implantation mechanical shear stress and acute ischemic trauma before a stable tissue capsule forms.

To systematically characterize these tissue responses and resolve temporal discrepancies, a semi-quantitative scoring system was implemented to grade the histopathological features across all explanted cohorts. Evaluated metrics included capsule thickness, density of inflammatory infiltrate, abundance of foreign body giant cells (FBGCs), fibrosis scoring, vascularization density, and local necrosis. Each parameter was graded on a scale of 0 to 3 (0 = absent, 1 = mild, 2 = moderate, 3 = severe). Our scoring revealed that while the acute mechanical elements (necrosis drops from grade 2 to 0 and hemorrhage diminishes from grade 2 to 0–1) resolve completely with increasing duration of residence, the overall chronic inflammatory infiltrate exhibits a biphasic or heterogeneous character rather than a linear decline. Specifically, early samples (1–2 years) display high cellularity dominated by neutrophils and active macrophages (inflammation grade 2–3). In intermediate years (3–6 years), the tissue exhibits a relative stabilization and capsule organization (inflammation grade 1–2). However, in long-term explants (8–14 years), a pronounced perifocal lymphocytic and macrophagic infiltrate re-emerges or persists (inflammation grade 2–3) in localized regions, as clearly observed in our 8-year sample (Figure 4a). This localized persistence of active inflammation alongside mature fibrotic tissue explains why a pronounced perifocal infiltrate can persist even after 8 to 14 years due to ongoing immune activation. As is well known, the presence of a foreign body in tissues invariably elicits an inflammatory response to some degree, driven by a state of “frustrated phagocytosis” where cells cannot engulf the massive filaments, and any pathology in the body merely amplifies this process, as observed in certain images. However, the complete absence of necrotic zones in ultra-long-term explants confirms that once the connective tissue capsule matures, it successfully cushions the surrounding anatomy and ensures excellent long-term clinical tolerance.

### 3.6. Gas Chromatography Results

Using gas chromatography–mass spectrometry (GC-MS), an attempt was made to answer this question by identifying compounds formed at the implant–biological tissue interface. A wide variety of isolated compounds attracted attention; however, no temporal dependence (as in histological studies) was observed.

To enhance clarity and facilitate analysis, the identified compounds have been organized by their chemical classification and the source matrix from which they were obtained, as shown in Table 4. This categorization differentiates between components originating from the native mesh material, the adjacent biological tissue, and external substances, including potential contaminants from processing or laboratory activities.

Gas chromatographic analysis results of both PP endoprostheses and surrounding biological tissues revealed substantial quantities of hydroxy acids and fatty acid esters, along with traces of acetamide. Acetamide, used as a polymer plasticizer, belongs to hazard class 2 according to the WHO classification [45] and is a potential human carcinogen. Furthermore, as explicitly highlighted in Table 4, the regular identification of phthalate species (such as benzyl butyl phthalate in the mesh control and dibutyl or di-n-octyl phthalate in the explants) must be interpreted with caution. These compounds frequently occur as ubiquitous laboratory contaminants introduced through plastic processing equipment, storage vessels, or analytical solvents, rather than representing true in vivo polymer degradation products. In certain cases, the presence of specific compounds may be associated with patients’ use of pharmaceutical preparations; therefore, such substances were naturally excluded from consideration in this study.

The presence of arachidonic acid in all variants of PP meshes and across all time intervals deserves particular attention. We hypothesize that the local tissue response, from the perspective of potential PP mesh influence, may be explained by the localized metabolic and mechanical activity of adipocytes. Thus, based on histological assessment (Figure 4), pronounced deformation of adipocytes was noted at the direct implant contact zones. Upon mechanical compression and inflammatory stimulation, adipocytes release fatty acids and glycerol via lipolysis. In turn, the carboxyl groups of fatty acids and hydroxyl groups of glycerol readily participate in various reactions, particularly with amide groups, which might theoretically interface with downstream metabolic pathways.

It is important to highlight that our study proposes a connection between the mechanical disruption of adipocytes and the upregulation of arachidonic acid. This connection is presented strictly as a qualitative, biologically plausible mechanistic hypothesis rather than a demonstrated causal relationship. While GC-MS screening assesses the overall extractable chemical profile, and histology provides insights into structural geometry, a direct quantitative assay measuring the real-time metabolic flux of this system, or the direct production of downstream inflammatory eicosanoids responsible for clinical pain, has not been conducted. Consequently, no definitive causality regarding patient symptom development or direct tissue inflammation can be inferred from these screening results. It should be noted that the concentration of released substances is extremely low—10^−3^ to 10^−5^%. It is worth noting that, although the analyzed meshes did not exhibit consistent evidence of bulk physicochemical or mechanical degradation, our findings cannot rule out alternative failure pathways. Clinical recurrence is inherently multifactorial, involving an intricate interplay of surgical technique, mesh positioning, mechanical compliance, and patient-specific comorbidities. Because comprehensive surgical and patient clinical metadata were not available for this cohort, a definitive cause of recurrence cannot be established. Therefore, our data suggest that while surface-confined changes are present, attributing the primary driver of surgical failure exclusively to bulk PP mesh degradation is not supported by these analytical parameters.

## 4. Conclusions

Investigation of polypropylene mesh endoprostheses explanted after surgical interventions due to recurrent postoperative hernias demonstrated that the physical, mechanical, and physicochemical properties of the endoprostheses remained consistent overall, though marked directional and individual sample variability was evident. Based on our analytical parameters, no clear evidence of progressive chemical oxidation of the polymer matrix was detected under the conditions used.

Virtually all polypropylene monofilaments constituting mesh endoprostheses undergo crack formation. Based on systematic software-assisted cross-sectional image metrics, the crack depth ranges from 3% to 7% of the filament diameter and does not significantly affect the overall strength properties of the endoprostheses. The micro-cracking and superficial delamination observed through electron microscopy are discussed as localized consequences of environmental stress cracking related to host foreign body interactions, rather than as definitive drivers of overall mechanical structural failure. While most explants retained substantial tensile strength exceeding standard physiological thresholds, directional vulnerabilities were observed (e.g., one sample displaying row strength at 13.6 N/cm). Due to simultaneous baseline variations in manufacturer design, density, and knitting parameters across the clinical cohort, mechanical discrepancies cannot be explicitly assigned to in vivo degradation alone, highlighting the combined influence of structural architecture and patient-specific remodeling.

Histological and chemical screenings revealed a persistent, low-grade foreign body reaction alongside localized indicators of mechanical adipocyte compression, such as free arachidonic acid. However, given the screening nature of these profiles and the lack of explicit surgical and patient metadata, these localized structural and biochemical transformations are presented without direct clinical correlation. Ultimately, a balanced interpretation of our findings shows that while the analyzed meshes exhibit superficial micro-cracking and ongoing localized host tissue reactions, they lack consistent evidence of bulk physicochemical or mechanical degradation. Since clinical hernia recurrence is inherently multifactorial and heavily dependent on specific surgical techniques, mechanical aspects, and patient characteristics—none of which were covered in this structural baseline study—the definitive root causes of clinical recurrence cannot be determined solely from these material evaluations.

## Figures and Tables

**Table 1:** Characteristics of explanted polypropylene mesh endoprostheses. *b* is endoprosthesis thickness, mm; *M_s_* is surface density of endoprosthesis, g/m^2^; *d* is diameter of filament used in endoprosthesis, mm. Weight classification categories: lightweight (35–50 g/m^2^) (samples 2, 8), medium-weight (50–80 g/m^2^) (samples 1, 4, 5, 6, 9, 10, 12, 13), and heavyweight (≥80 g/m^2^) (samples 2, 7, 11).

No.	In Vivo Duration [years]	*B* [mm]	*M_s_* [g/m^2^]	*d* [mm]
1	0	0.54	54	0.13
2	1	0.41	50	0.10
3	1	0.65	110	0.17
4	2	0.54	78	0.16
5	3	0.62	72	0.18
6	3	0.44	63	0.14
7	4	0.49	140	0.12
8	5	0.35	47	0.10
9	6	0.57	78	0.13
10	8	0.57	74	0.15
11	11	0.53	106	0.14
12	13	0.54	72	0.13
13	14	0.53	80	0.11

**Table 2:** Thermophysical properties of mesh endoprosthesis samples at different explantation intervals. *τ* is in vivo exposure time; *T_cryst_* is crystallization temperature, °C; *T_melt_* is melting temperature, °C; *H* is enthalpy, J/g, *DC* is degree of crystallinity, %.

No.	*Τ* [years]	*T_cryst_* [°C]	*T_melt_* [°C]	*H* [J/g]	*DC* [%]
1	0	107	162	90	65.2
2	1	105	162	94	68.1
4	2	105	163	91	65.9
5	3	104	165	93	67.4
9	6	105	164	80	58.0
10	8	105	166	113	81.9
11	11	105	162	78	56.5
13	14	106	164	90	65.2

**Table 3:** Deformation-strength properties of explanted endoprostheses. *P_loop_* is breaking load along loop column; *P_row_* means breaking load along loop row; ε*_loop_* is relative elongation at break along loop column; ε*_row_* describes relative elongation at break along loop row.

No.	In Vivo Duration [years]	*P_loop_* [N/cm]	*P_row_* [N/cm]	ε*_loop_* [%]	ε*_row_* [%]
1	0	46.9 ± 4.69	44.2 ± 4.42	113	100
2	1	46.9 ± 4.69	43.5 ± 4.35	113	110
3	1	76.0 ± 7.60	28.0 ± 2.8	82	211
4	2	96.8 ± 9.68	60.1 ± 6.01	52	102
5	3	27.9 ± 2.79	22.7 ± 2.27	130	91
6	3	32.1 ± 3.21	26.3 ± 2.63	108	116
7	4	39.1 ± 3.91	19.5 ± 1.95	158	180
8	5	31.1 ± 3.11	13.6 ± 1.36	74	84
9	6	48.5 ± 4.85	25.6 ± 2.56	134	133
10	8	26.6 ± 2.66	26.6 ± 2.66	158	101
11	11	48.4 ± 4.84	30.0 ± 3.00	52	102
12	13	41.2 ± 4.12	28.1 ± 2.81	73	103
13	14	42.4 ± 4.24	26.1 ± 2.61	123	146

**Table 4:** Classification of compounds detected via GC-MS analysis.

Chemical Category	Detected in Control PP Mesh (Without Tissue)	Detected in Surrounding Biological Tissue Explants	Potential Source/Contamination Note
Plasticizers and Structural Mesh Additives	Acetamide; Tridecyl ester of 2-propenoic acid; pentadecyl ester	Acetamide; benzyl methacrylate	Polymer manufacturing residues/compounding agents.
Phthalate Esters (Suspected Contaminants)	Benzyl butyl phthalate; mono-2-ethylhexyl ester of benzenedicarboxylic acid	Di-n-octyl phthalate; dibutyl phthalate; 1,4-benzenedicarboxylic acid	Likely exogenous laboratory or processing artifacts (e.g., plastics, solvents, or storage).*
Fatty Acids and Lipids	Hexadecanoic acid ester	Dodecanoic acid; tetradecanoic acid (and ester); cis-9-hexadecenoic acid (and ester); trans-9-hexadecenoic acid; hexadecanoic acid (and ester); N-pentadecanoic acid; heptadecanoic acid; oleic acid; octadecanoic acid; octadecadienoic acid ester; 9,12-octadecadienoic acid; cis-9-octadecenoic acid; trans-9-octadecenoic acid; 11-cis-octadecenoic acid; 9-tetradecenoic acid; cholesterol; glycerol; squalane; octadecanol; hexadecanol	Endogenous tissue matrix, membrane lipids, and metabolic lipolysis products.
Polyunsaturated Fatty Acids (PUFAs)	None	Arachidonic acid; cis-5,8,11-eicosatrienoic acid; cis-11,14-eicosadienoic acid; cis-13-eicosenoic acid; cis-7,10,13-docosatetraenoic acid	Cellular membranes/localized metabolic pathways.
Amino Acids and Nitrogenous Compounds	None	Alanine; L-valine; L-leucine; L-isoleucine; L-proline; glycine; L-threonine; L-methionine; phenylalanine; 5-oxo-L-proline; urea; 2,4-dioxopyrimidine; 1,3-dihydro-2,4-pyrimidinedione; 9H-hydroxypurine; 1-methyl-4-hydroxy-1H-imidazol-2-amine	Tissue degradation, protein turnover, and proteinaceous encapsulation.
Carbohydrates and Derivatives	Cyclododecane homolog; cyclopentanecarboxylic acid	TMS-ether of D-glucose; 1,2,3,5-tetrakis-O-arabinofuranose; 2,3,4,5-tetrakis-O-arabinose; 1,2,3,5-tetrakis-O-arabinopyranose; tetrakis-1-deoxyglucose; 2,3,4,5,6-pentakis-O-D-glucose (and linear); 2,3,4,5,6-pentakis-O-D-altrose; pentakis-O-D-glucopyranose; 1,2,3,5,6-pentakis-D-galactofuranose; 1,2,3,4,6-pentakis-galactopyranose; 1,2,3,4,5,6-hexakis-O-myo-inositol	Cellular glycoconjugates, extracellular matrix sugars, or derivatization byproducts.
Organic Acids and Short-Chain Esters	None	Propanoic acid ester; 2-methylpropanoic acid ester; 2-hydroxypropanoic acid; 2-hydroxy-2-propenoic acid; D-hydroxypropanoic acid; 2-hydroxybutanoic acid; 2-ethylhexanoic acid; 3-hydroxybutyric acid; nonanedioic acid; octanoic acid; nonanoic acid; decanoic acid; 2,3-dihydroxypropanoic acid	Intermediate tissue metabolic products.
Amides and Miscellaneous Organic Residues	None	N,N′-bis-urea; N,N-dimethylethanolamine; hexadecanenitrile; oleanonitrile; N,N-ethylbutylamide of formic acid; octyl ether; tripropylene glycol; dodecanamide; hexadecanamide; octadecanamide; 9-octadecenamide; ethanol, 2-phenoxy-	Trace manufacturing residues, lipid–amide conjugations, or ambient chemical artifacts.
Vitamins and Antioxidants	None	α-tocopherol (vitamin E); 7,9-di-tert-butyl-oxopyro(4,5)-deca-6,9-diene-2,8-dione	Endogenous antioxidants/processing stabilizers.

## Data Availability

The original contributions presented in this study are included in the article. Further inquiries can be directed to the corresponding author.
